# Cannabis use is associated with reduced prevalence of non-alcoholic fatty liver disease: A cross-sectional study

**DOI:** 10.1371/journal.pone.0176416

**Published:** 2017-04-25

**Authors:** Adeyinka Charles Adejumo, Samson Alliu, Tokunbo Opeyemi Ajayi, Kelechi Lauretta Adejumo, Oluwole Muyiwa Adegbala, Nnaemeka Egbuna Onyeakusi, Akintunde Micheal Akinjero, Modupeoluwa Durojaiye, Terence Ndonyi Bukong

**Affiliations:** 1 Department of Medicine, University of Massachusetts Medical School, Worcester, Massachusetts, United States of America; 2 Biomedical Engineering & Biotechnology Program, University of Massachusetts Lowell, Lowell Massachusetts, United States of America; 3 Department of Medicine, Maimonides Medical Center, Brooklyn, New York, United States of America; 4 Johns Hopkins Medicine, Howard County General Hospital, Columbia, Maryland, United States of America; 5 Public Health Program, University of Massachusetts Lowell, Lowell, United States of America; 6 Department of Medicine, Englewood Hospital and Medical Center, Englewood, New Jersey, United States of America; 7 Department of Pediatrics, Bronx-Lebanon Hospital Center, Bronx, New York, United States of America; 8 Department of Maternal and Child Health, University of Alabama at Birmingham, Birmingham, Alabama, United States of America; 9 INRS-Institut Armand-Frappier, Institut National de la Reserche Scientifique, Laval, Quebec, Canada; Medizinische Fakultat der RWTH Aachen, GERMANY

## Abstract

Cannabis use is associated with reduced prevalence of obesity and diabetes mellitus (DM) in humans and mouse disease models. Obesity and DM are a well-established independent risk factor for non-alcoholic fatty liver disease (NAFLD), the most prevalent liver disease globally. The effects of cannabis use on NAFLD prevalence in humans remains ill-defined. Our objective is to determine the relationship between cannabis use and the prevalence of NAFLD in humans. We conducted a population-based case-control study of 5,950,391 patients using the 2014 Healthcare Cost and Utilization Project (HCUP), Nationwide Inpatient Survey (NIS) discharge records of patients 18 years and older. After identifying patients with NAFLD (1% of all patients), we next identified three exposure groups: non-cannabis users (98.04%), non-dependent cannabis users (1.74%), and dependent cannabis users (0.22%). We adjusted for potential demographics and patient related confounders and used multivariate logistic regression (SAS 9.4) to determine the odds of developing NAFLD with respects to cannabis use. Our findings revealed that cannabis users (dependent and non-dependent) showed significantly lower NAFLD prevalence compared to non-users (AOR: 0.82[0.76–0.88]; p<0.0001). The prevalence of NAFLD was 15% lower in non-dependent users (AOR: 0.85[0.79–0.92]; p<0.0001) and 52% lower in dependent users (AOR: 0.49[0.36–0.65]; p<0.0001). Among cannabis users, dependent patients had 43% significantly lower prevalence of NAFLD compared to non-dependent patients (AOR: 0.57[0.42–0.77]; p<0.0001). Our observations suggest that cannabis use is associated with lower prevalence of NAFLD in patients. These novel findings suggest additional molecular mechanistic studies to explore the potential role of cannabis use in NAFLD development.

## Introduction

Cannabis is the most abused substance which is still illicit in most countries globally [1]. Besides alcohol and tobacco, it is the most widely used drug for recreational purposes [2]. It is currently estimated that between 119–224 million people worldwide [1], and over 22 million individuals in the United States of America (USA), aged 12 years and above use cannabis [2]. With the recent rise in cannabis legalization across different states in the USA, this number is expected to grow even further. While cannabis use (CU) is associated with increased prevalence of disorders such as schizophrenia [3], cyclical hyperemesis syndrome [4], and pulmonary diseases [5], it has also been linked to a reduction in obesity [6] and diabetes mellitus (DM) [7–9]. The prevalence of these disorders is expected to be impacted as CU increases over the coming years.

Non-Alcoholic Fatty Liver Disease (NAFLD) is a metabolic disorder characterized by excess fat accumulation in the liver and is the most common liver disease in the world [10]. About one-third of US adults have been diagnosed with NAFLD [11,12]. While excessive fat accumulation in the liver has previously been thought to be harmless, studies including ours, have demonstrated that NAFLD can progress to steatohepatitis, fibrosis, cirrhosis and even hepatocellular carcinoma [13,14]. By 2020, it is estimated that progressive NAFLD would become the leading cause of advance liver disease requiring liver transplantation in patients [15]. Obesity and DM are among the known risk factors for NAFLD [16]. Since CU has been associated with reduced prevalence of both obesity [6] and DM [7–9], we surmised that CU might also have modulatory effects on the prevalence of NAFLD.

Our study was aimed at testing the hypothesis that CU is associated with reduced prevalence of NAFLD given its suppressive effect against obesity and diabetes in humans. Our novel studies revealed that CU was associated with reduced prevalence of NAFLD in patients. However, co-commitment NDCU and dependent abusive alcohol consumption, which can independently induce progressive fatty liver disease, negated the reduced prevalence of NAFLD observed in cannabis-only users.

## Materials and methods

### Data source and study population

We conducted a population-based case-control study to determine the relationship between CU and NAFLD. We used data from the Healthcare Cost and Utilization Project Nationwide Inpatient Sample (HCUP-NIS) database, which is produced by the Agency for Healthcare Research and Quality (AHRQ) [17]. HCUP-NIS is the largest inpatient database, comprising of over 3000 hospitals sampled from more than 40 states representing over 7 million annual hospital discharges in the U.S.A. For this study, we used the 2014 HCUP-NIS data was collected from January 1^st^ to December 31^st^, 2014. The data contained 7,071,762 patient discharge records. Each discharge record contained up to 30 different clinical diagnoses using the International Classification of Diseases, Ninth Edition, Clinical Modification (ICD-9-CM). The clinical discharge records also included patient demographics, insurance type, clinical procedures, hospital costs, comorbidities, and hospital characteristics. The HCUP-NIS has been used in numerous studies to provide reliable estimates of conditions within the U.S.A [18–22]. Given that the HCUP-NIS is a completely de-identified and publicly available data, no Institutional Review Board approval was required. We evaluated clinical records for patients 18–90 years old (n = 5,950,391).

### Characterization of outcomes and exposure variables

Our primary outcome was a diagnosis of NAFLD, defined using ICD-9-CM codes (Table 1). Code 571.8 represents, "other chronic nonalcoholic liver disease". This code is for nonalcoholic liver diseases and includes both Non-Alcoholic Steatohepatitis (NASH) and NAFLD. Recent studies have used this code as an indicator for NAFLD diagnosis [23,24]. Our primary exposure variable was based on current CU, which was stratified into two main groups to investigate dose-dependent CU relationships in NAFLD, namely; dependent CU (DCU, ICD-9-CM codes 304.3x) and non-dependent CU (NDCU, ICD-9-CM codes 305.2x). According to ICD-9-CM, substances coded as CU include patient use of Indian hemp, marijuana and other varieties of cannabis and cannabinoids.

**Table 1:** ICD-9-CM codes for identifying NAFLD, cannabis use (dependent and non-dependent), and other risk factors.

Clinical Condition	ICD-9-CM Codes
Non-Alcoholic Fatty Liver Disease	571.8
Cannabis Abuse	
Dependent:	304.3, 304.30, 304.31, 304.32, 304.33
Non-Dependent:	305.2, 305.20, 305.21, 305.22, 305.23
Tobacco Abuse	
Past	305.1
Current	V15.82
Diabetes Mellitus*	250.x
Dyslipidemia⁑	272.x
Alcohol Abuse	
Past	V11.3
Present	303, 303.0, 303.00, 303.01, 303.02, 303.03, 305.0, 305.00, 305.01, 305.02, 305.03
Overweight**	278.02
Obese†	278.00, 278.01, 278.03
Hypertension	401, 401.0, 401.1, 401.9
Metabolic Syndrome	277.7

* Includes Type 1 and Type II DM;

⁑ Includes unspecified disorders of lipoid metabolism.;

** Includes BMI from 25.0 to 29.9 Kg/m^2^;

^†^ Includes BMI from > = 30 Kg/m^2^

We focused on the following NAFLD associated risk factors: demographics (age, gender, race, socioeconomic status, insurance type) and patient specific conditions (hypertension, DM, dyslipidemia, metabolic syndrome, obesity, tobacco). All ICD-9-CM codes used in identifying patient conditions are presented in Table 1. Given increased prevalence of NAFLD among the middle age group, we categorized this group into three sub-groups: <40yrs, 40-60yrs, and >60 years. We additionally categorized racial origins into four sub-groups: Non-Hispanic Whites, Non-Hispanic Blacks, Hispanics, Others (Asians and Native-Americans). Based on the average annual household income of where the patient resides (zip code), we categorized socio-economic status into four groups: lowest quartile, second quartile, third quartile and highest quartile. Patient's gender was either male or female. We classified health insurance types into five sub-categories: Medicare, Medicaid, Private, Self-pay and Others (no charge, other government insurance, worker's compensation, miscellaneous).

Overweight (pre-obese) and obesity diagnosis included patient discharge records with a BMI of 25–29.9 and > = 30 kg/m^2^ respectively. Tobacco use was defined as having a history or current use of tobacco products; diabetes as having a diagnosis of DM (type I or type II), and alcohol use as having a history or current abusive use of alcohol. We defined hyperlipidemia as having a lipid metabolism disorder including hypercholesterolemia, hyperglyceridemia, hyperchylomicronemia, lipodystrophy, lipoprotein deficiencies, lipidoses, mixed hyperlipidemias and other disorders resulting in elevated plasma lipids. We defined hypertension as having a clinical diagnosis of essential hypertension.

Drinking levels of alcohol is defined clinically following the National Institute on Alcohol Abuse and Alcoholism (NIAAA) and the Substance Abuse and Mental Health Services Administration (SAMHSA) standards following guidelines from the U.S. Department of Health and Human Services and U.S. Department of Agriculture.

### Data analysis

All statistical analyses were performed using the Statistical Analysis System (SAS) [Release Version 9.4, SAS Institute Inc, Cary, NC, US]. We characterized patient discharge records with descriptive statistics and reported the mean and standard deviation (SD) for continuous variables and the percentages for categorical variables. We used Chi-squared (X^2^) test to analyze differences between the sub-groups of categorical variables. We additionally used SURVEYFREQ procedure in SAS, with the STRATA and WEIGHT statements to account for the HCUP-NIS sampling method.

We evaluated the crude's odds ratio (COR) by running a univariate binary logistic regression to assess the association between NAFLD (present/absent) and various risk factors. These factors included CU (DCU and NDCU), age, tobacco, DM, hypertension, obesity, race, dyslipidemia, metabolic syndrome, insurance status and household income. Next, we evaluated the adjusted odds ratio (AOR) by running a multivariate logistic regression between NAFLD and cannabis use for the above-listed risk factors as covariates. For regression analysis, we used the SURVEYLOGISTIC procedure in SAS, with the STRATA and WEIGHT statements to account for the HCUP-NIS sampling method.

Using the final adjusted model described above, we further assessed for effect modification by introducing the interaction term between CU and the each of the other risk factors for NAFLD individually. For the identified risk factors with a significant interaction with CU, we then ran a stratified analysis and measured the effect of CU on NAFLD prevalence.

A pvalue of <0.05 was considered statistically significant for all our statistical tests used in this study.

## Results

### Study population in relation to cannabis use

Of total of 5,950,391 patient discharge records, aged 18 years and above analyzed, we found that 5,833,812 (98.0%) were non-cannabis users, 103,675 (1.74%) were NDCU and 12,904 (0.22%) were DCU (Table 2).

**Table 2:** NAFLD, socio-demographic characteristics, and other risk factors among patient discharge records (18 years and above) classified by marijuana use (dependent and non-dependent) and non-use.

	Non-cannabis users, % (n = 5,833,812, 98.0%)	Non-dependent cannabis users, % (n = 103,675, 1.74%)	Dependent cannabis users, % (n = 12,904, 0.22%)	p-value
**Demographics**				
**NAFLD (present)**	0.91	0.77	0.38	<0.0001
**Age group (years)**				<0.0001
18–40	24.2	56.7	66.1	
41–60	25.1	35.5	29.8	
>60	50.7	7.8	4	
**Gender**				<0.0001
Male	40.7	62.1	66.7	
Female	59.3	37.9	33.3	
**Race**				<0.0001
White	68.7	54.4	59.2	
Black	14.5	31.2	24.7	
Hispanic	10.7	9.5	9.9	
Asian and Others*	6.2	4.9	6.2	
**Insurance**				<0.0001
Medicaid	47	17.7	16.3	
Medicare	17.4	43.7	43.3	
Private	28.3	20.2	23.6	
Self-Pay	4.1	12.9	11.2	
Others⁑	3.3	5.5	5.5	
**Household Median Income**†				<0.0001
First Quartile	29.5	42.6	36.6	
Second Quartile	27.7	26.5	26.4	
Third Quartile	23	18.7	20.3	
Fourth Quartile	19.9	12.3	16.7	
**Comorbidities**				
**Hyperlipidemia**				<0.0001
No	69.6	87.5	91.7	
Yes	30.4	12.5	8.3	
**Hypertension**				<0.0001
No	60.9	74	81.1	
Yes	39	25.9	18.9	
**Diabetes**				<0.0001
No	74.6	86.4	92.1	
Yes	25.4	13.6	7.9	
**Overweight**				<0.0001
No	99.7	99.5	99.4	
Yes	0.3	0.5	0.6	
**Obesity**				<0.0001
No	86.7	90.8	92.9	
Yes	13.3	9.2	7.1	
**Alcohol**				<0.0001
No	94.5	71.2	59.9	
Yes	5.6	28.8	40.1	
**Tobacco**				<0.0001
No	71.8	39.9	45.1	
Yes	28.2	60.1	54.9	
**Metabolic Syndrome**				<0.0001
No	99.8	99.9	99.9	
Yes	0.17	0.11	0.08	

* Asians, Pacific Islanders and Native Americans;

⁑ No charge, other government, Indian Health Service, Worker's compensation, other miscellaneous;

^†^Annual income stratified by residence zip-code, 1st quartile:$1-$39,999, 2nd quartile:$40,000-$50,999, 3rd quartile:$51,000-$65,999, 4th quartile:$66,000+

The unadjusted prevalence of NAFLD was lower among CU when compared to non-users (DCU:0.38%, NDCU:0.77% vs. non-users:0.91%). NDU and DCU were more likely to be between the ages 18–40 years and 40–60 years compared to >60 years old. Cannabis users (DCU and NDCU) were more likely to be male, white, have Medicare insurance, and be in the lowest quartile of income. When compared to non-cannabis users, DCU and NDCU were less likely to have hyperlipidemia (8.3%, 12.5% vs. 30.4%) and diabetes (7.9%, 13.6% vs. 25.4%). Though they were slightly more likely to be overweight (0.62%, 0.49% vs. 0.32%), they were however less likely to be obese (7.7%, 9.7% vs. 13.6%) or have metabolic syndrome (0.08%, 0.11% vs. 0.17%). DCU and NDCU were more likely to abuse alcohol (40.1%, 28.8% vs. 5.6%) and tobacco (54.9%, 60.1% vs. 28.2%).

### Relationship between NAFLD with cannabis use and other risk factors for NAFLD in humans

The development of NAFLD is associated with numerous risk factors. To determine how CU relates to other risk factors of NAFLD development, we estimated the crude odds ratio (COR) for NAFLD with CU, and similarly with other known risk factors of NAFLD (Table 3). We found that CU was associated with 20% reduced prevalence of NAFLD (AOR: 0.80[0.75–0.87]). When compared to non-users, the prevalence of NAFLD was respectively 68% and 15% less among DCU and NDCU (AOR: 0.42[0.32–0.55] and 0.85[0.79–0.92]). We observed a dose-response effect of cannabis use as DCU had 47% less prevalence of NAFLD when compared to NDCU (AOR 0.53[0.39–0.72]). After adjusting for the potential confounding risk factors listed in Table 3 in our statistical modeling, the association between cannabis use and NAFLD did not change significantly (<10%). These observations suggested that the effect of CU on NAFLD might be mediated through alternative factors other than those evaluated by our modeling. It is worth mentioning that all known factors for NAFLD were included in our final model.

**Table 3:** Crude and adjusted odds ratio for NAFLD among risk factors for NAFLD among patient.

NAFLD (present)	Crude OR	Adjusted† OR
**Cannabis use**		
Dependent use	0.42 (0.32–0.55)*	0.45 (0.34–0.61)*
Non-dependent use	0.85 (0.79–0.91)*	0.84 (0.78–0.91)*
Non-use	Ref	Ref
**Age group (years)**		
18–40	Ref	Ref
41–60	2.45 (2.39–2.51)*	1.56 (1.51–1.60)*
>60	1.17 (1.15–1.20)*	0.91 (0.87–0.94)*
**Gender**		
Male	Ref	Ref
Female	0.90 (0.88–0.91)*	1.05 (1.03–1.06)*
**Race**		
White	Ref	Ref
Black	0.63 (0.61–0.65)*	0.56 (0.54–0.58)*
Hispanic	1.28 (1.25–1.32)*	1.29 (1.25–1.32)*
Asian and Others**	0.96 (0.93–0.99)*	1.02 (0.98–1.06)
**Insurance**		
Medicaid	0.64 (0.63–0.65)*	0.67 (0.66–0.69)*
Medicare	0.74 (0.72–0.75)*	0.76 (0.74–0.78)*
Private	Ref	Ref
Self-Pay	1.06 (1.02–1.10)*	1.04 (0.99–1.07)
Others⁑	0.77 (0.74–0.81)*	0.78 (0.74–0.82)*
**Household Median Income**††		
First Quartile	0.93 (0.91–0.95)*	0.89 (0.87–0.92)*
Second Quartile	0.97 (0.95–0.99)*	0.92 (0.89–0.95)*
Third Quartile	0.99 (0.96–1.02)	0.94 (0.92–0.97)*
Fourth Quartile	Ref	Ref
**Hyperlipidemia**		
No	Ref	Ref
Yes	1.52 (1.50–1.55)*	1.19 (1.17–1.22)*
**Hypertension**		
No	Ref	Ref
Yes	1.52 (1.50–1.55)*	1.19 (1.17–1.22)*
**Diabetes**		
No	Ref	Ref
Yes	2.27 (2.23–2.31)*	1.85 (1.81–1.88)*
**Overweight**		
No	Ref	Ref
Yes	1.83 (1.61–2.08)*	2.12 (1.86–2.41)*
**Obesity**		
No	Ref	Ref
Yes	3.99 (3.86–4.12)*	3.13 (3.03–3.24)*
**Alcohol**		
No	Ref	Ref
Yes	1.81 (1.76–1.86)*	1.93 (1.87–1.99)*
**Tobacco**		
No	Ref	Ref
Yes	1.23 (1.21–1.25)*	1.05 (1.03–1.07)*
**Metabolic Syndrome**		
No	Ref	Ref
Yes	6.76 (6.22–7.36)*	2.72 (2.49–2.97)*

*p-value<0.0001;

^†^Adjusted for all other risk factors in our study;

**Asians, Pacific Islanders and Native Americans;

⁑ No charge, other government, Indian Health Service, Worker's compensation, other miscellaneous;

^††^Annual income stratified by residence zip-code, 1st quartile:$1-$39,999, 2nd quartile:$40,000-$50,999, 3rd quartile:$51,000-$65,999, 4th quartile:$66,000+

All reported estimates were after adjustment for potential confounders (Table 3). When compared to the 18-40-year age group, individuals between the 40–60 and >60-year age groups had 56% higher and 9% lower prevalence of NAFLD (AOR: 1.56[1.51–1.60] and 0.91[0.87–0.94]) respectively. The prevalence of NAFLD was 5% and 26% elevated among females and hypertensives (AOR: 1.05[1.03–1.06] & 1.26[1.23–1.28]), 20% higher among those with hyperlipidemia (AOR: 1.19[1.17–1.22]), 85% higher among DM patients (AOR: 1.85[1.81–1.88]), 120% higher among the pre-obese (AOR: 2.12[1.86–2.41]), 330% higher among the obese (AOR: 3.13[3.02–3.24]), and 170% higher among those with metabolic syndrome (AOR: 2.72[2.49–2.97]). The prevalence of NAFLD was also 5% and 90% higher among people with a past or a current history of tobacco and alcohol use respectively (AOR: 1.05[1.03–1.07] and AOR: 1.93[1.87–1.99]). Compared to non-Hispanic Whites, non-Hispanic Blacks had 45% reduced prevalence of NAFLD (AOR: 0.56[0.55–0.58]), while Hispanics have 30% increased prevalence of NAFLD (AOR: 1.29[1.26–1.33]). There was no statistical difference in the prevalence of NAFLD among Asians/Other races and Whites (AOR: 1.02[0.98–1.06]). Compared to those on Private health insurance, patients on Medicaid, Medicare and others (including uninsured) had 33%, 25%, and 18% less prevalence of NAFLD (AOR: 0.67[0.66–0.69], 0.76[0.74–0.78], and 0.78[0.74–0.82]). Compared to the upper-income bracket, every other lower income quartile had a reduced prevalence of NAFLD. This decrease trended down with household wealth, from the richest to the poorest quartile. When compared to the richest (fourth) quartile, the prevalence of NAFLD was 4%, 8% and 11% less in the third, second and first (poorest) quartile (AOR: 0.94[0.92–0.97], 0.92[0.89–0.95], and 0.89[0.87–0.92]).

### Sensitivity and post-hoc analysis

Among all the risk factors analyzed by our study, age, obesity, alcohol use, and hyperlipidemia showed significant statistical interactional association with cannabis in its association with NAFLD (Table 4). We found that the effects of CU on NAFLD was heterogeneous across the age groups (p = 0.0011). NDCU was only associated with a reduced prevalence of NAFLD in individuals 40–60 years (Interaction AOR: 0.77 (0.72–0.84), p<0.0012). DCU was associated with a 2 to 3-fold reduced prevalence of NAFLD among individuals 18–40 and 40–60 years but not among those >60 years, where there was no difference compared to non-users (Interaction AOR: 3.1 (1.77–5.43), p = 0.043). Obesity was associated with about 2 to 3-fold elevation in the prevalence of NAFLD so that CU (DCU and NCDU) was no longer associated with a lower prevalence of NAFLD among the obese (Interaction OR: 2.3(1.68–3.10), p = 0.0073).

**Table 4:** Modification of the effect of cannabis on NAFLD by age, obesity, alcohol use, hyperlipidemia.

Modifying factor	Sub-group analysis with AOR (95% CI)		
		Dependent user	Non-dependent user
**Overall effect**		0.45 (0.34–0.61)*	0.85 (0.79–0.92)*
**Age group**	18–40 yr	0.35 (0.22–0.56)*	0.95 (0.85–1.06)
	40–60 yr	0.51 (0.34–0.77)*	0.74 (0.66–0.82)*
	>60 yr	1.07 (0.40–2.88)	1.07 (0.83–1.38)
**Obesity**	Obese	0.85 (0.53–1.36)	0.90 (0.78–1.04)
	Not Obese	0.35 (0.24–0.50)*	0.83 (0.76–0.90)*
**Alcohol use**	Use Alcohol	0.40 (0.27–0.61)*	0.69 (0.61–0.78)*
	No Alcohol use	0.51 (0.34–0.78)*	0.96 (0.87–1.05)
**Hyperlipidemia**	Have Hyperlipidemia	0.97 (0.60–1.60)	0.92 (0.80–1.07)
	No Hyperlipidemia	0.34 (0.24–0.49)*	0.82 (0.75–0.90)*

*p-value: <0.0001

Alcohol use was associated with a 90% increase in NAFLD prevalence (Table 3, AOR: 1.93(1.87–1.99)). Alcohol use, however, showed a differential modulatory effect on NAFLD prevalence amongst CU. Among both alcohol consumers, DCU was associated with a lower prevalence of NAFLD. Among NDCU, there was a lower prevalence of NAFLD in alcoholics compared to non-alcoholics (Interaction AOR: 0.72 (0.66–0.78), p<0.0001). To further explore the effects of CU-alcohol use interactions, we categorized alcohol use into two-subgroups: non-dependent users, and dependent users, to respectively constitute moderate and severe consumption. Statistical analysis with these variable adjustments revealed that NDCU was associated with a 53% lower prevalence of NAFLD among non-dependent alcohol consumers (AOR: 0.57(0.48–0.68)) but not among dependent (AOR: 0.95(0.87–1.05)). DCU, however, was associated with a 70% and 55% reduction in NAFLD prevalence among non-dependent and dependent consumers of alcohol (AOR: 0.30(0.11–0.79) & 0.45(0.28–0.70)).

Given that NAFLD patients who take alcohol could also develop Alcoholic Steatohepatitis (ASH), we completely excluded patients with any past or current history of alcoholic use (n = 358,507) from our study, and re-estimated the crude and adjusted OR for developing NAFLD among the remaining subjects (n = 5,591,884). NDCU and DCU were respectively associated with 21% and 66% reduced prevalence for NAFLD (COR: 0.79(0.73–0.87) & 0.34(0.23–0.52)) [Table 5]. After accounting for other risk factors of NAFLD, NDCU was only associated with 2% reduced prevalence and was no longer statistically significant, unlike DCU which was associated with a significant 48% reduced prevalence of NAFLD (AOR: 0.52(0.34–0.80)).

**Table 5:** Crude and adjusted odds ratio for NAFLD after excluding patients with any past or present history of alcohol use.

Cannabis use	Crude OR (95% CI)	Adjusted† OR (95% CI)
Dependent use	0.34 (0.23–0.52)*	0.52 (0.34–0.80)*
Non-dependent use	0.79 (0.73–0.87)*	0.98 (0.89–1.07)
Non-use	Ref	Ref

*p-value<0.0001;

^†^Adjusted for all other risk factors in our study

## Discussion

**Fig 1:**
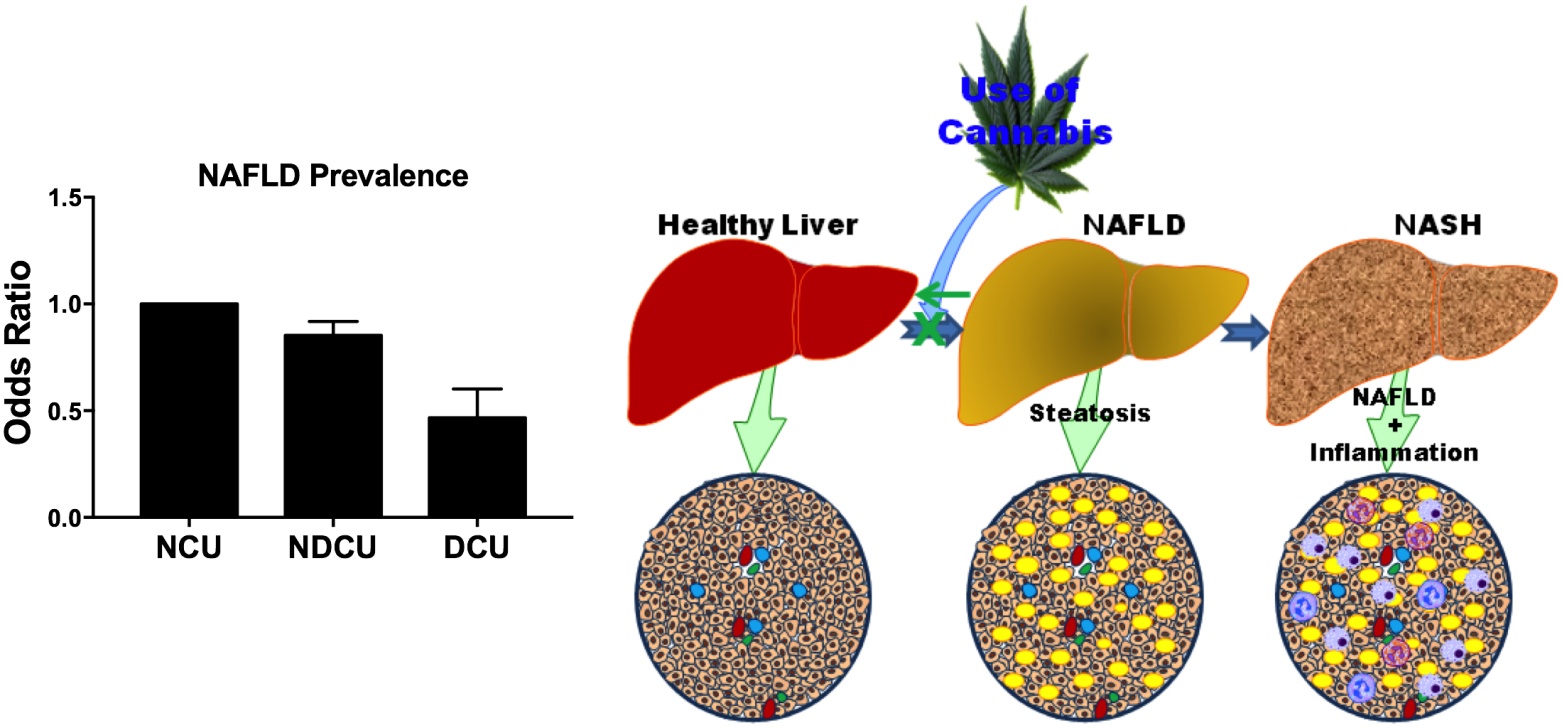
Cannabis use is associated with reduced non-alcoholic fatty liver disease. Dependent and Non-Dependent Cannabis Use (DCU & NDCU) are associated with reduced NAFLD when compared to non-cannabis users (NCU). These observations suggest that dependent cannabis use suppresses or reverses the development and progression of NAFLD to advance liver disease (non-alcoholic steatohepatitis [NASH]). Illustrated schematics made use of some motifolio templates (www.motifolio.com).

Our study explored the 2014 NIS data set to evaluate the association between CU and NAFLD among discharged patients aged 18 years and above in the USA. We observed a strong dose-dependent reduction in the prevalence of NAFLD with cannabis use (Fig 1) suggesting that cannabis use might suppress or reverse NAFLD development. Additionally, increased prevalence of NAFLD was associated with known factors including age 40–60 years, female gender, hyperlipidemia, hypertension, alcohol, diabetes, and metabolic syndrome. We observed racial differences in NAFLD, whereby non-Hispanic Whites and Hispanics had higher odds for disease compared to non-Hispanic Blacks. Patients with public health insurance (Medicare and Medicaid) had a lower prevalence of NAFLD compared to privately insured and uninsured individuals (Health insurance and type of insurance relate broadly to access and quality of health care provided to patients respectively). This novel revelations contrast commonly held notions and suggest that individuals on government health insurance might have a reduced prevalence of a chronic disorder such as NAFLD due to improved emphasis on preventive services since the implementation of the Affordable Care Act [25,26].

**Fig 2:**
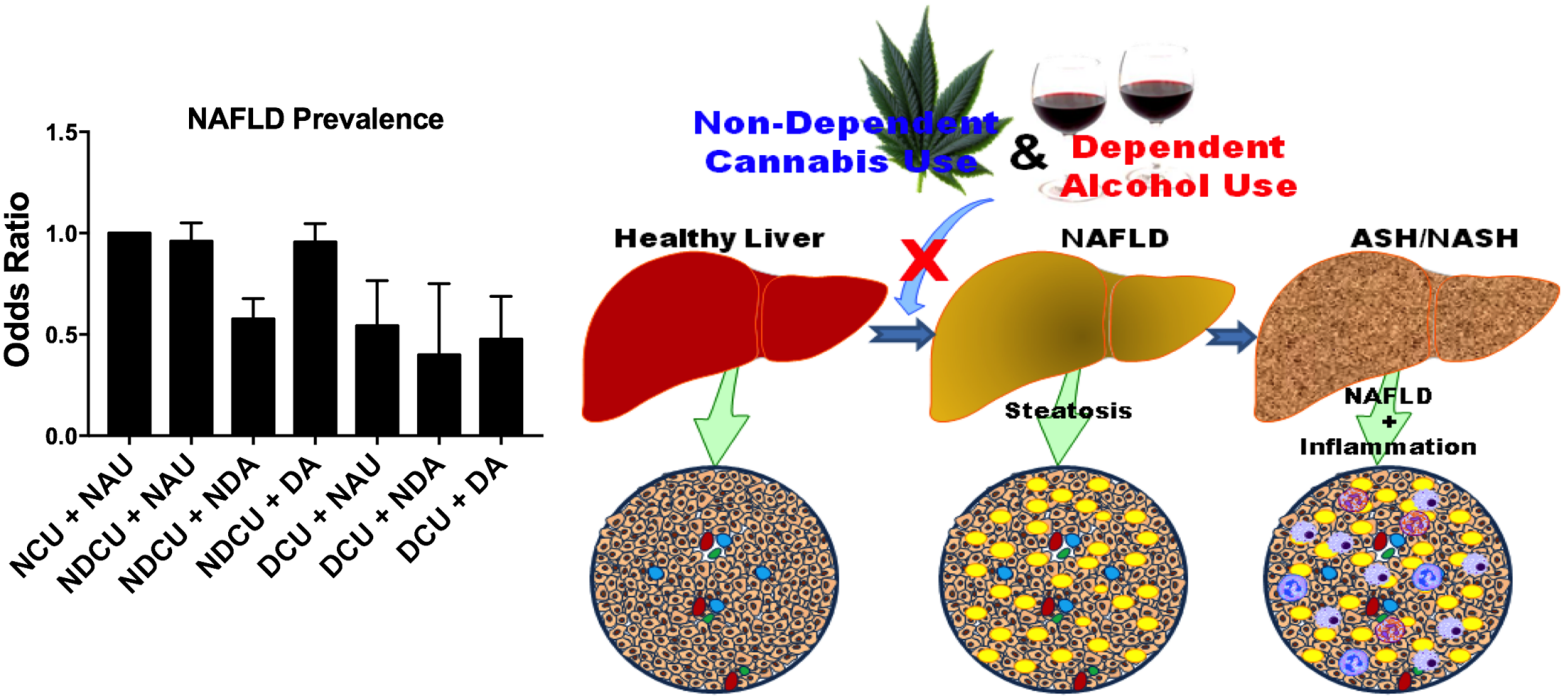
Dependent alcohol use abolishes reduced NAFLD prevalence observed in non-dependent cannabis users. Bar graph described from the leftmost to the rightmost: Taking non-cannabis users/non-alcohol users (NCU+NAU) as the reference group, non-dependent (moderate) cannabis use/non-alcohol use (NDCU+NAU) caused a slight reduction in NAFLD prevalence, though not statistically significant. Non-dependent (moderate) alcohol use among NDCU (NDCU+NDA) resulted in a reduced NAFLD prevalence, though this protection was lost with dependent (abusive) alcohol use (NDCU+DA). However dependent (high volume) cannabis use (DCU) was always associated with a reduction in NAFLD prevalence among all categories of alcohol users: non-alcohol (DCU+NAU), moderate/non-dependent alcohol users (DCU+NDA) and copious/dependent alcohol users (DCU+DA). Illustrated schematics made use of some motifolio templates (www.motifolio.com).

We found that alcohol use was associated with a higher prevalence of NAFLD. Though CU reduced the prevalence of NAFLD in patients, only non-dependent alcohol use maintained some of this beneficial health effect (Fig 2). NDCU only had lower odds for NAFLD among non-dependent alcohol individuals, unlike DCU which maintained lower odds for NAFLD irrespective of the volume of alcohol use (non-dependent & dependent) (Fig 2). Studies have reported that DCU more likely consume cannabis at a larger quantity and frequency than NDCU [27,28]. Therefore, with higher consumption of cannabis, DCU might have a higher dose and longer duration of cannabis acting on the hepatic cannabinoid receptors, and offering a protective effect when compared to NDCU. A recent study has indeed shown that cannabis act directly on hepatic cells and protects against liver disease [29]. Taken together, these observations suggest that chronic dependent alcohol negated the beneficial effects of NDCU in modestly reducing the prevalence of NAFLD in patients. Our CU-non-dependent alcohol user interaction observations suggests that moderate alcohol intake, irrespective of the volume of cannabis used, might have beneficial effects in reducing the prevalence of NAFLD. Our novel observations are somehow in concert with published findings which demonstrated that moderate alcohol consumption provided some beneficial effects in NAFLD patients [28].

CU is negatively associated with many risk factors of NAFLD characterized by reduced prevalence of DM [8,9,29,30], with overweight/obesity [6], and hyperlipidemia [7]. Cannabis might act on fatty deposits in obese tissues through omega-3 (n-3) fatty acids [31]. For example, preclinical trials have revealed that endogenously produced cannabinoids mediate the ability of (n-3) fatty acids in reducing ectopic fats deposits and fatty inflammation [30]. A meta-analysis in humans further confirms that dietary (n-3) supplementation resulted in a reduction in steatosis and improved liver enzyme profile [31]. Another study demonstrated that CU resulted in reduced fasting insulin, insulin resistance, smaller waist circumference and higher levels of HDL [32]. Taken together, these observations suggest that cannabis use has modulatory effects on NAFLD risk factors.

Given that the effects of CU on NAFLD persisted even after accounting for the other risk factors for NAFLD and the proposed mediators (obesity, hyperlipidemia, DM) in our statistical modeling, we surmise that cannabis probably acts on the liver via alternate mediators and mechanisms. In addition to other ingredients, cannabis contains tetrahydrocannabinol (THC) and cannabidiol (CBD) [33,34]. THC and CBD act on CB-1 and CB-2 receptors [35]. While THC has more effect on the CB-1, CBD act preferentially on CB-2. CB-1 has been shown to be more pro-inflammatory and psychotropic, unlike CB-2, which is more anti-inflammatory and non-psychotropic [35,36]. Pre-clinical studies have demonstrated that hepatic CB-1 receptors up-regulate hepatic fatty acid production and participate in diet-induced obesity [37]. Hepatic CB-1 receptors also promote liver fibrosis in both murine and human subjects [38]. In contrast, CB-2 agonists can suppress obesity, steatohepatitis and can additionally protect the liver from ischemic reperfusion injury. Further, CB-2 agonist decrease hepatic immune cells infiltration, kupffer cell activation, oxidative stress, hepatic injury and fibrogenesis [39–42]. Taken together, our novel observations and previous findings suggest that CB-2 agonist and hemp which has a higher CBD might offer more protection from NAFLD [43,44].

The association between age and NAFLD remains controversial based on published literature. While some studies showed that the prevalence of NAFLD rises with age [45–48], many have reported otherwise [49–52]. It is viewed that since the risk factors for NAFLD including DM, obesity and hyperlipidemia all increase with age, NAFLD prevalence should equally follow this trend. Given that numerous studies have however demonstrated that NAFLD prevalence and age follows an "inverted U shaped curve" [53], we reason that this could be due to two factors: One could be that with increasing age, there is relatively more visceral fat deposition [54] and reduced subcutaneous and hepatic fat deposits [55]. This argument might however be faulted because visceral fat correlates with hepatic fat and NAFLD [56]. Another reason could be because individuals with NAFLD also have a higher cardio-metabolic risks, they may die early from other associated diseases, resulting in fewer elderly patients with NAFLD in the study population[57,58].

Strikingly, low-income patients had the lowest prevalence of NAFLD compared to patients in the higher income bracket. Being in a lower median household income corresponds to having a lower socioeconomic status (SES) or poverty. It is well established that low SES is associated with obesity, hypertension, diabetes, hyperlipidemia [59–61], which are all predisposing factors for NAFLD. It would be expected that the association of these conditions with low SES would result in a higher NAFLD among the low income population. We did additional posthoc analysis on our data, where we built statistical models for each of the aforementioned four conditions. Besides hyperlipidemia were the odds for NAFLD was reduced and further decreased with reducing income, the odds for obesity, hypertension and diabetes was higher and trended upwards as the median income level reduced (S1 Table). Despite three of the four risk factors for NAFLD associated with decreasing income, the odds for NAFLD was lower with reducing income both in our crude and adjusted models. The reasons for this new observation remain unclear, but suggest that hyperlipidemia might be a more important predisposing risk factor for NAFLD development. A pediatric study revealed no association between maternal poverty levels and developing NAFLD in children [62]. We suspect that the slight reduction in odds for NAFLD among individuals of low socioeconomic status might be due to a higher non-exercise related physical activity from engaging in more manual occupations. Given that the liver is responsible for energy (gluconeogenisis and lipogenesis) production and storage in high fed states, and the body first depletes hepatic energy stores before resulting to extra-hepatic reserves during manual activities. Individuals with more blue-collar jobs might be more likely to expend the hepatic stores and prevent/reduce long term hepatic fat accumulation. A recent study demonstrated that NAFLD subjects undergoing any form of physical activity experience significant reduction in their hepatic fat deposits even without any change in total body weight [63–65]. A more comprehensive meta-analysis revealed that different forms of exercise, ranging from low-impact to vigorous exertion, might all be beneficial for NAFLD [66–68]. More prospective studies are however needed to determine the reasons for this observation.

### Strengths and limitations

Our study is limited by its cross-sectional study design [69]. These include recall bias in reporting exposures, not well-established sensitivity and specificity of ICD-9-CM codes for cannabis use. Indeed, the ICD-9-CM code does not differentiate the particular type of cannabis plant used. We, however, expect the recall bias, and less specificity and sensitivity of ICD-9-CM cannabis codes to have a similar chance of occurring among those with NAFLD as well as those without NAFLD. With this imprecision in the coding, our results would likely be biased towards the null, that is no association, and the true effect of CU on NAFLD might be underestimated.

The lack of temporal observations in cross-sectional studies does inhibit our ability to draw direct causal relationships between CU and NAFLD. Failure to measure and or record other possible confounding factors might limit our inferences on the effects of CU and NAFLD. To strengthen our observations, we reviewed recent literature to identify additional risk factor for NAFLD which were not retrievable from the NIS dataset [16].

Different strains of the cannabis plant also contain different ratios of anti-inflammatory CBD and pro-psychotic/pro-inflammatory THC agents [34,36,43,44]. For example, hemp, cannabis oil, hash oil, sativa, and indica all have different contents of CBD and THC and the various contents of these active ingredients could not be assessed from the data set analyzed.

Despite the shortcomings of cross-sectional studies, our novel observations are necessary given the increased legalization of cannabis in the USA and increased prevalence of NAFLD globally. The fact that we used the NIS data archives, which is a very reliable large population-based database, allows for under-reported outcomes, such as cannabis use and NAFLD with good statistical power.

## Conclusions

To the best of our knowledge, this is the first population-based cross-sectional study of hospitalized patients to explore the associations between CU and NAFLD. Our analyses revealed a strong relationship between cannabis use and reduced prevalence of NAFLD in patients. Due to our inability to draw direct causation effects from our cross-sectional studies, we suggest prospective basic and human studies to decipher the mechanistic details of how the various active ingredients in cannabis modulate NAFLD development.

## Supporting information

S1 Table Adjusted odds ratio for having a diagnosis of NAFLD among different income levels with obesity, hypertension, diabetes and hyperdilipemia. *p-value<0.0001; ** Annual income stratified by residence zip-code, 1st quartile:$1-$39,999, 2nd quartile:$40,000-$50,999, 3rd quartile:$51,000-$65,999, 4th quartile:$66,000+.
(DOCX)
