# Supplementary material for: Cannabis use is associated with reduced prevalence of non-alcoholic fatty liver disease: A cross-sectional study
Source: PLoS One. 2017 Apr 25;12(4):e0176416. doi: 10.1371/journal.pone.0176416 (PMC5404771; doi:10.1371/journal.pone.0176416)
Supplement: S1 Table — *p-value<0.0001; ** Annual income stratified by residence zip-code, 1st quartile:$1-$39,999, 2nd quartile:$40,000-$50,999, 3rd quartile:$51,000-$65,999, 4th quartile:$66,000+. (DOCX) [file pone.0176416.s001.docx]

| **Median Household Income**** | **Obesity** | **Hypertension** | **Diabetes** | **Hyperlipidemia** |
| --- | --- | --- | --- | --- |
| First vs. Fourth Quartile | 1.15 (1.14-1.16)* | 1.14 (1.13-1.15)* | 1.46 (1.45-1.47)* | 0.89 (0.84-0.95) |
| Second vs. Fourth Quartile | 1.17 (1.16-1.18)* | 1.09 (1.09-1.10)* | 1.33 (1.32-1.34)* | 0.96 (0.91-1.02) |
| Third vs. Fourth Quartile | 1.14 (1.13-1.15)* | 1.04 (1.04-1.05)* | 1.21 (1.20-1.21)* | 0.96 (0.91-1.02) |
